# The Capsid (ORF2) Protein of Hepatitis E Virus in Feces Is C-Terminally Truncated

**DOI:** 10.3390/pathogens11010024

**Published:** 2021-12-26

**Authors:** Takashi Nishiyama, Koji Umezawa, Kentaro Yamada, Masaharu Takahashi, Satoshi Kunita, Mulyanto, Isao Kii, Hiroaki Okamoto

**Affiliations:** 1 Division of Virology, Department of Infection and Immunity, Jichi Medical University School of Medicine, 3311-1 Yakushiji, Shimotsuke-shi 329-0498, Japan; kentaro-y@cc.miyazaki-u.ac.jp (K.Y.); mtaka84@jichi.ac.jp (M.T.); 2 Laboratory for Drug Target Research, Integrated Bioscience Division, Institute of Agriculture, Shinshu University, 8304 Minamiminowa, Kamiina 399-4598, Japan; ikii@shinshu-u.ac.jp; 3 Department of Biomedical Engineering, Institute for Biomedical Sciences, Shinshu University, 8304 Minamiminowa, Kamiina 399-4598, Japan; koume@shinshu-u.ac.jp; 4 Laboratory of Veterinary Public Health, Department of Veterinary Sciences, Faculty of Agriculture, University of Miyazaki, Miyazaki 889-2192, Japan; 5 Center for Experimental Medicine, Jichi Medical University School of Medicine, 3311-1 Yakushiji, Shimotsuke-shi 329-0498, Japan; sakunita@jichi.ac.jp; 6 West Nusa Tenggara Hepatitis Laboratory, Mataram 83231, Indonesia; mulyanto@unram.ac.id; 7 Immunobiology Laboratory, Faculty of Medicine, University of Mataram, Mataram 83125, Indonesia

**Keywords:** hepatitis E virus, nonenveloped infectious virion, fecal sample, truncated ORF2 protein

## Abstract

The hepatitis E virus (HEV) is a causative agent of hepatitis E. HEV virions in circulating blood and culture media are quasi-enveloped, while those in feces are nonenveloped. The capsid (ORF2) protein associated with an enveloped HEV virion is reported to comprise the translation product of leucine 14/methionine 16 to 660 (C-terminal end). However, the nature of the ORF2 protein associated with fecal HEV remains unclear. In the present study, we compared the molecular size of the ORF2 protein among fecal HEV, cell-culture-generated HEV (HEVcc), and detergent-treated protease-digested HEVcc. The ORF2 proteins associated with fecal HEV were C-terminally truncated and showed the same size as those of the detergent-treated protease-digested HEVcc virions (60 kDa), in contrast to those of the HEVcc (68 kDa). The structure prediction of the ORF2 protein (in line with previous studies) demonstrated that the C-terminal region (54 amino acids) of an ORF2 protein is in flux, suggesting that proteases target this region. The nonenveloped nondigested HEV structure prediction indicates that the C-terminal region of the ORF2 protein moves to the surface of the virion and is unnecessary for HEV infection. Our findings clarify the maturation of nonenveloped HEV and will be useful for studies on the HEV lifecycle.

## 1. Introduction

Hepatitis E is acute hepatitis, a generally self-limiting and rarely fatal disease, with a lethality of 0.5–3% among young adults; however, this rate reaches 20–30% in pregnant women [1,2]. This form of hepatitis is caused by hepatitis E virus (HEV) infection via the fecal–oral route, from polluted water in developing countries [1,2]. Furthermore, in developed countries, hepatitis E has been increasingly reported as a zoonotic food-borne transfusion-associated, or organ-transplantation-associated, disease [3,4], and chronic cases can occur in immunocompromised individuals, such as those receiving organ transplants, patients with hematological malignancy, and HIV-infected patients [5,6,7].

HEV belongs to the *Hepeviridae* family, which is classified into two genera: *Orthohepevirus* and *Piscihepevirus* [8]. The genus, *Orthohepevirus,* is divided into four species (A–D). The *Orthohepevirus A* species includes eight different HEV genotypes (1–8). Genotypes 1–4 and 7 are known to infect humans [9], while genotypes 5 and 8 are reported to infect monkeys and are suggested to carry a risk for zoonotic infection [10,11]. Apart from the *Orthohepevirus A* species, recent studies have documented zoonotic infections of humans with rat HEV, belonging to the *Orthohepevirus C* species [12,13].

HEV has an approximately 7.2-kilobase (kb) single-stranded positive-sense RNA genome. This viral genome encodes three proteins: a nonstructural polyprotein required for RNA replication (ORF1); a capsid protein of the HEV virion (ORF2); and a small multifunctional protein with key functions in particle assembly and release (ORF3) [14,15]. The ORF2 protein contains three distinct domains: S (shell, amino acids [aa] 118–313); M (middle, aa 314–453); and P (protruding, aa 455–end) [16]. The S domain is composed of jelly roll-like β-sheets; the M domain is tightly linked to the S domain and is located at the surface; and the P domain dimerizes, forming protruding spikes [17,18,19]. The ORF2 protein is also secreted as dimerized and glycosylated protein [20]. The ORF3 protein is localized at the lipid membrane of the enveloped virion [21]. Both the ORF2 and ORF3 proteins are translated from a bicistronic subgenomic RNA, approximately 2.2 kb in length [22].

HEV is secreted into the culture media and the circulating blood as an enveloped HEV with a host-derived lipid membrane and ORF3 protein [23], but it is shed into feces as a nonenveloped HEV, without the lipid coat or ORF3 protein [21]; thus, it has been declared a “quasi-enveloped” virus [24]. Takahashi et al. [25] reported that detergent- and protease-treated enveloped HEV lost the ORF3 protein and lipid membrane and displayed a buoyant density of 1.27 g/mL, similar to fecal nonenveloped HEV, suggesting that the treated enveloped HEV can be regarded as a nonenveloped HEV.

Recent reports have revealed the nature of the virion-associated ORF2 protein: it has an intact C-terminal end, is nonglycosylated [20], and is translated from the second methionine 16 (M16; ORF2^c^) [26] and/or starts with L14 (ORF2_i_) [20]. However, the precise nature of the fecal nonenveloped HEV virion-associated ORF2 protein is poorly understood.

In the present study, we show that the ORF2 protein constituting both the fecal HEV virion and the detergent (sodium deoxycholate [DOC])- and protease-treated enveloped HEV are C-terminally truncated. We predicted the protease digestion sites in the truncated ORF2 (ORF2tr) protein. In addition, our structure prediction suggests the digested region of the ORF2tr protein in the fecal HEV virion. Our results reveal that the ORF2 protein in the fecal HEV virion differs from that of the enveloped HEV virion with an intact ORF2 C-terminus.

## 2. Results

### 2.1. The C-Terminal Region of the HEV Capsid (ORF2) Protein Is Truncated in Human Feces

**Figure 1:**
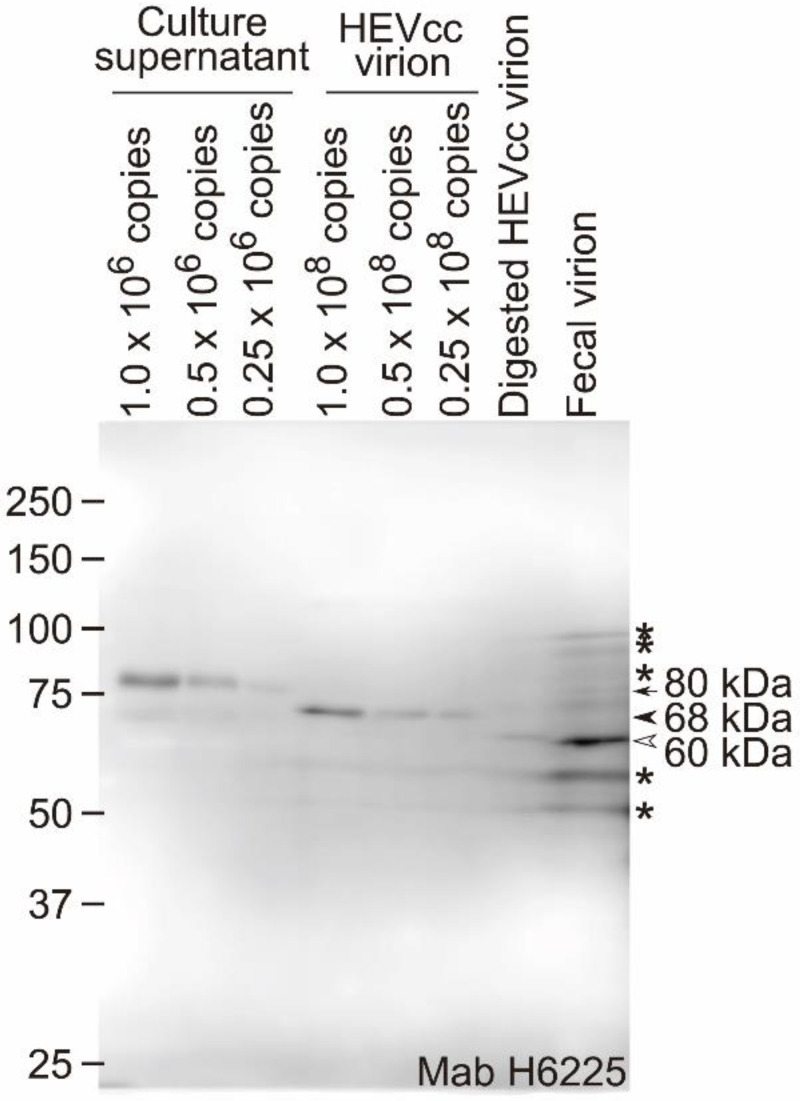
Molecular size of the ORF2 protein in feces. The difference in the molecular sizes of the ORF2 protein of the human HEV virion in the culture supernatant and the human fecal sample. The culture supernatant (2.5, 1.3, and 0.63 µL of culture supernatant, containing 1.0, 0.5, and 0.25 × 10^6^ copies of HEV, respectively, per lane), the ultracentrifuged HEVcc virions in the culture supernatant (1.0, 0.5, and 0.25 × 10^8^ copies of HEV, derived from 250, 125, and 63 µL, respectively, of culture supernatant/lane) of the HEV-producing cells, the 0.5% DOC- and 0.5% trypsin-treated HEVcc virions in the culture supernatant (0.5 × 10^8^ copies of HEV, derived from 125 µL of culture supernatant: digested virion), and the ultracentrifuged nonenveloped HEV virion (1.0 × 10^8^ copies of HEV: fecal virion) in the fecal sample from an HEV patient were subjected to SDS-PAGE, and were detected with the anti-HEV mouse monoclonal antibody H6225. Arrow, secreted glycosylated and dimerized ORF2 protein; arrowhead, ORF2 protein associated with enveloped HEV; hollow arrowhead, truncated ORF2 associated with nonenveloped HEV; asterisk, nonspecific signals.

When subjected to sodium dodecyl sulfate-polyacrylamide gel electrophoresis (SDS-PAGE) and immunoblotting, the secreted glycosylated ORF2 proteins and the virion-associated ORF2 proteins in the culture supernatant of the genotype 3b HEV (JE03-1760F)-producing PLC/PRF/5 cells were detected in approximately 80- and 68-kDa forms, respectively (Figure 1), consistent with previous reports [20,26]. However, in contrast to the ORF2 proteins in the culture supernatant (glycosylated and dimerized) and HEVcc, the ORF2 protein of the HEV virion in feces was detected in a 60-kDa form (Figure 1). Similar to the ORF2 protein in the fecal HEV, the detergent (0.5% DOC)-treated protease (0.5% trypsin)-digested HEVcc virion-associated ORF2 protein was also detected in a 60-kDa form (Figure 1). Both of these ORF2 proteins are described as “ORF2tr” in this manuscript.

Xing et al. [17] reported that the C-terminal region of the ORF2 protein is located at the surface of the native virion-sized *T = 3* VLP of HEV, consisting of an HEV RNA fragment and the 180 ORF2 proteins without the N-terminal 13 residues. Other studies also report that the C-terminal regions of the ORF2 proteins were located at the surface of VLP [16,18,27]. Furthermore, our previous study indicated that the antihuman HEV ORF2, Mab (H6225), was able to capture the HEV virions in feces and the DOC-treated trypsin-digested HEVcc virion and inhibited the infection of cells with fecal HEV and de-enveloped HEVcc in vitro [28].

**Figure 2:**
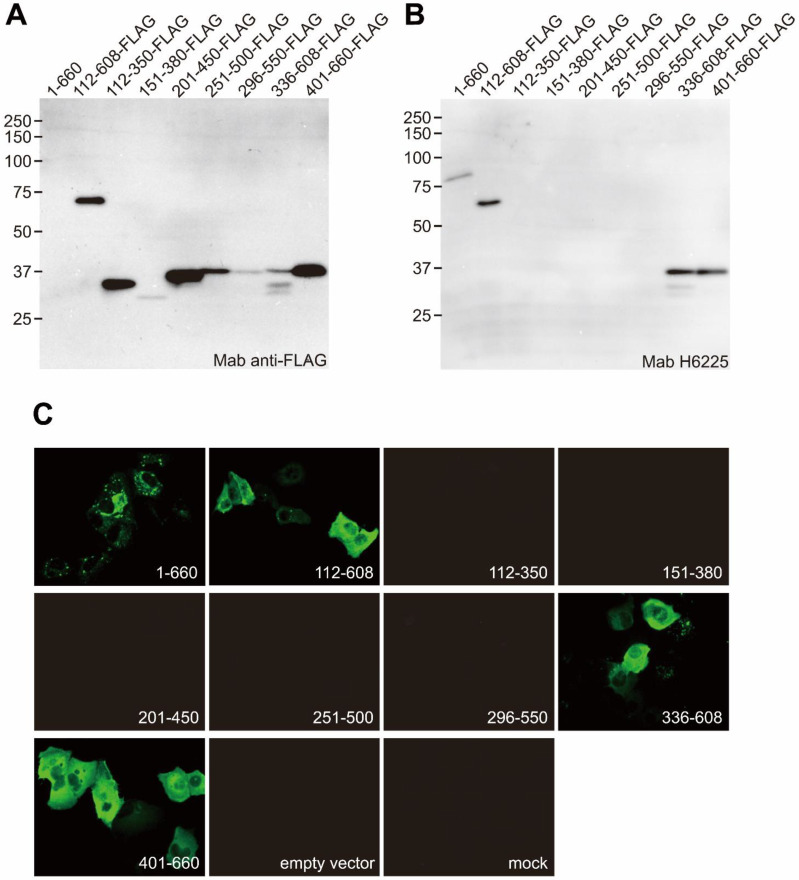
The epitope mapping of H6225, the anti-HEV ORF2 mouse monoclonal antibody. The ORF2 full length and FLAG-tagged deletion constructs were transfected to PLC/PRF/5 cells, lysed, and subjected to SDS-PAGE. Blotted membranes were probed with anti-FLAG (**A**) H6225 anti-HEV mouse monoclonal antibodies (**B**). (**C**) An immunofluorescence analysis with H6225 Mab. The cells transfected for the constructs were stained with H6225 Mab. The recognition site of the H6225 Mab was mapped between G551-A608 of the ORF2 protein.

In the present study, we found that the H6225 Mab recognizes the G551-A608 sequence of the ORF2 protein by epitope mapping (Figure 2). Therefore, to confirm that the protease digestion in the HEV virion occurs at the C-terminal region of the ORF2 protein, we constructed a recombinant HEV JE03-1760F/P10 genome (P10_ORF2-FLAG) to produce HEV-like particles with the C-terminally FLAG-tagged ORF2.

**Figure 3:**
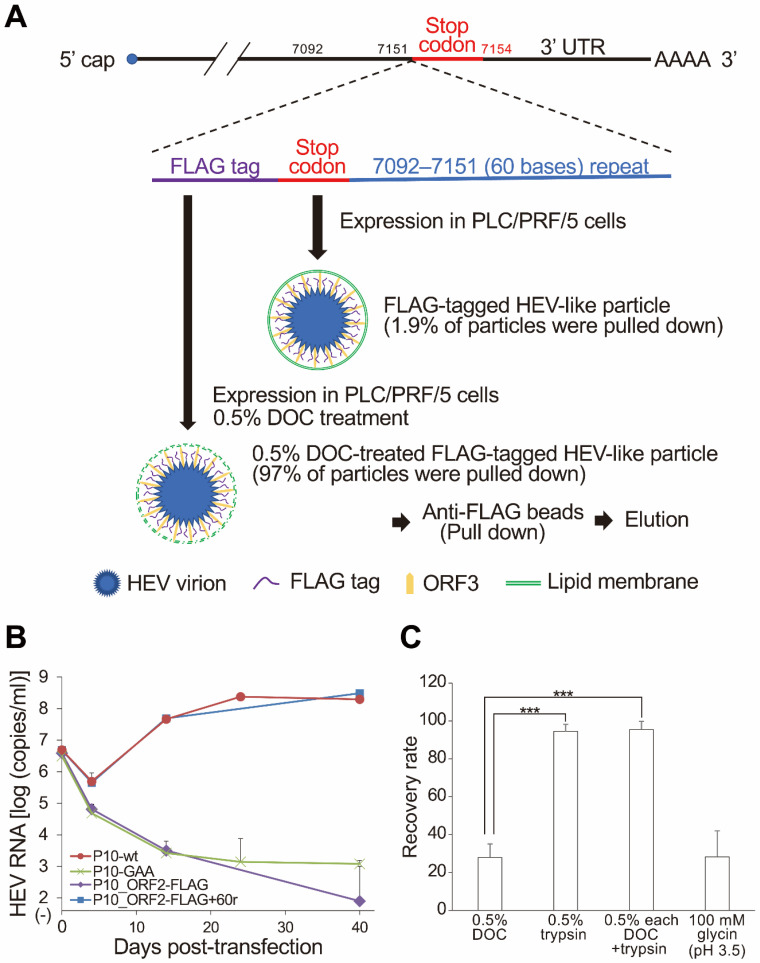
Pull-down of FLAG-tagged HEV-like particle and its elution with trypsin. (**A**) The schematic image of the FLAG-tagged HEV (genotype 3, JE03-1760F/P10)-like particle construct, expression, and pull-down with anti-FLAG magnetic beads. The FLAG (DYKDDDDK) tag was inserted into the C-terminal end of the ORF2-protein-coding region. In addition, the 7092–7151 (60 bases) repeat of the JE03-1760F/P10 genome (essential region for viral replication) was inserted between the stop codon after the inserted FLAG tag sequence and the authentic stop codon for the ORF2 coding sequence. The expressed HEV-like particle contained a FLAG tag at the C-terminal end of the ORF2 protein. The culture supernatant containing the FLAG-tagged HEV-like particles (P10_ORF2-FLAG-60r) was treated with 0.5% DOC and captured with anti-FLAG magnetic beads and then eluted, with the HEV RNA genome in the eluent subsequently measured. (**B**) The growth curve of FLAG-tagged HEV-like particles. The synthesized RNA genomes, the FLAG-tagged ORF2 coding JE03-1760F/P10 RNA, with (P10_ORF2-FLAG + 60r) or without (P10_ORF2-FLAG) the 7092–7151 (60 bases) repeat, and with P10-wt RNA as a positive control and P10-GAA RNA as a negative control for growth, were transfected to PLC/PRF/5 cells. The growth of the wild-type and modified HEV-like particles was monitored with RT-qPCR and plotted (*n* = 3). Error bars represent the mean ±SD. (**C**) The captured FLAG-tagged HEV-like particles were eluted with 0.5% DOC, 0.5% trypsin, both 0.5% DOC and 0.5% trypsin, and 100 mM glycine (pH 3.0). Error bars represent the mean ± SD. *** *p* < 0.001 by an ANOVA with the Tukey–Kramer test (*n* = 3).

However, probably because the inserted FLAG sequence disrupted the essential RNA sequence for HEV genomic RNA replication [29,30,31], P10_ORF2-FLAG was not grown in the cultured cells. To solve this issue, we inserted the 7091–7151 sequence (60 bases) of the HEV JE03-1760/P10 genome, corresponding to the 3′-terminal end sequence of the ORF2 protein, between the stop codon after the FLAG tag coding sequence, and the authentic stop codon of the ORF2 coding sequence (Figure 3A). After transfection of PLC/PRF/5 cells with RNA transcripts of the modified FLAG-tagged JE03-1760F/P10 genome (P10_ORF2-FLAG + 60r), FLAG-tagged HEV-like particles were successfully produced and released into the culture media, similar to the parental HEV, JE03-1760F/P10 (P10-wt), and in contrast to the P10_ORF2-FLAG FLAG-tagged ORF2 coding sequence without the 7092–7151 (60 bases) repeat (Figure 3B). The obtained FLAG-tagged HEV-like particles were then pelleted down and treated with 0.5% DOC, before being pulled down with anti-FLAG M2 magnetic beads (binding efficiency: ≈97%, Figure 3A); this process resulted in the cell-culture-generated FLAG-tagged HEV-like particle, having the ORF2 protein with an uncleaved C-terminus.

The bound FLAG-tagged HEV-like particles were significantly more efficiently eluted by 0.5% trypsin digestion, with or without 0.5% DOC, compared to 0.5% DOC elution only (Figure 3C). This result indicates that the FLAG-tagged HEV-like particles were de-enveloped by DOC, and that the C-terminally FLAG-tagged ORF2 protein lost its C-terminal region with the FLAG tag after digestion by trypsin.

### 2.2. Protease Digestion Sites of the ORF2 Protein Associated with the HEV Virion

**Figure 4:**
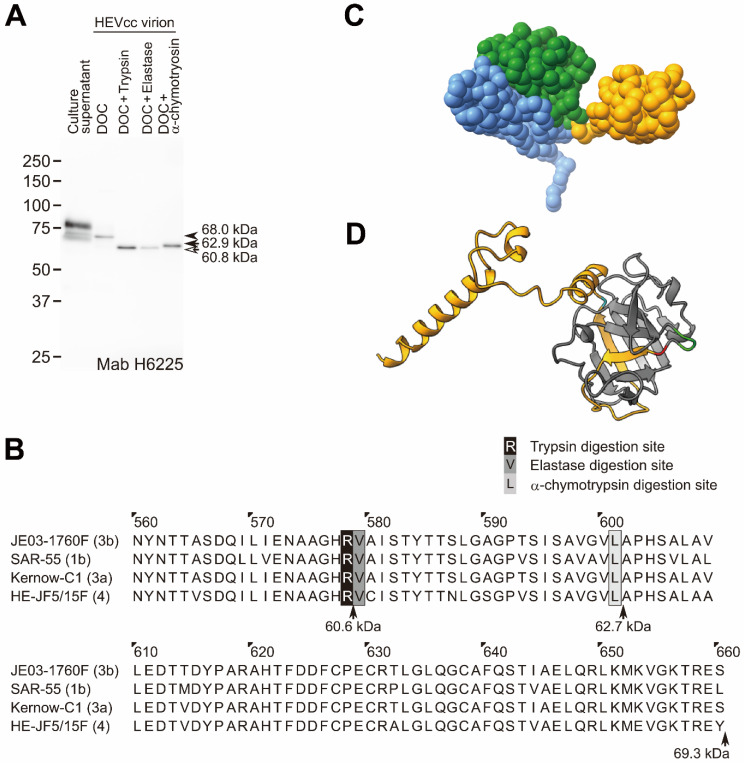
Protease digestion site in the ORF2 protein. Trypsin digests human HEVcc (JE03-1760F strain) virion between R578 and V579. (**A**) The culture supernatant (2.5 µL of culture supernatant containing 1.0 × 10^6^ copies of HEV/lane), the 0.5% DOC-treated, 0.5% DOC-treated, and 0.5% trypsin-digested; 0.5% DOC-treated and 0.5% elastase-digested; and 0.5% DOC-treated and 0.5% α-chymotrypsin-digested HEVcc virions (1.0 × 10^8^ copies each of ultracentrifuge-purified HEV derived from 250 µL of culture supernatant/lane) were subjected to SDS-PAGE, and detected with the anti-HEV mouse monoclonal antibody, H6225. Arrowhead, arrow, and hollow arrow indicate full-length ORF2, α-chymotrypsin-digested ORF2, and trypsin- or elastase-digested ORF2 proteins, respectively. Molecular weights were estimated with the size marker. (**B**) The alignment of 560–660 amino acids of the ORF2 protein sequences from the genotypes, 3b (JE03-1760F), 1b (SAR-55), 3a (Kernow-C1), and 4 (HE-JF5/15F). Arrows indicate the calculated molecular weights: M16-R578, 60.6 kDa; M16-L601, 62.7 kDa; M16-S660, 69.3 kDa. (**C**) The structure of the amino acids 118–06 of the ORF2 protein (3IYO). The S (shell, 118–313), M (middle, 314–453), and P (protruding, 455–end) domains are highlighted in blue, green, and orange, respectively. (**D**) The structure of the P domain and C-terminal region (P461-S660) of the genotype 3b HEV (JE03-1760F) ORF2 protein was predicted using the AlphaFold2 software program. The A574-G576, R578 residue, V579-S660, and L601 are highlighted in green, red, yellow, and cyan, respectively.

To test whether or not other typical proteases (α-chymotrypsin and elastase) secreted in the intestine digested HEV virion, we treated HEVcc virion with 0.5% DOC, and then 0.5% elastase, or 0.5% α-chymotrypsin (or with 0.5% trypsin as a control). After digestion with trypsin, elastase, and α-chymotrypsin, the molecular sizes of the ORF2 protein (originally 68.9 kDa) of the HEVcc virion changed to 60.8, 60.8, and 62.9 kDa, respectively, as estimated with a molecular size marker (Figure 4A).

To estimate the sites digested by trypsin, elastase, or α-chymotrypsin in the C-terminal region of the ORF2 protein, we aligned the ORF2 amino acid sequences (aa 560–660) of four representative HEV strains (genotype 3b, JE03-1760F, which is used in this study; genotype 1b, SAR-55; genotype 3a, Kernow-C1; genotype 4, HE-JF5/15F). On the basis of the reported protease digestion sites [32,33,34], R578, A579, and L601 were estimated to be the trypsin, elastase, and α-chymotrypsin digestion sites of the ORF2 protein, respectively (Figure 4B). The R578 residue of the ORF2 protein was conserved among 180 HEV strains of genotypes 1–8 (Supplementary Table S1), suggesting that the ORF2 protein of fecal HEV was digested at R578 (furthest away from the C-terminus among the three typical proteases in the intestine), and probably lacked the 88 C-terminal amino acid residues (V579-S660 fragment), consistent with the length of the DOC-treated trypsin-digested ORF2 protein (ORF2tr) observed on SDS-PAGE (Figure 4A).

**Figure 5:**
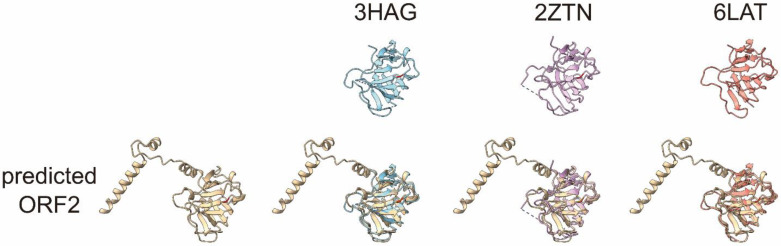
The superimposed image of the AlphaFold2-predicted ORF2 and previously reported ORF2 protein structures. The AlphaFold2-predicted ORF2 protein (amino acids 461–660) structure and the previously reported ORF2 protein structure, 3HAG (amino acids 461–605 [16]), 2ZTN (amino acids 461–606 [18]), and 6LAT (amino acids 461–605 [27]) were superimposed using the UCSF ChimeraX software program, ver. 1.1. The R578 residue is highlighted in red. Dotted line represents disordered region.

To visualize the possible digestion sites, we next predicted the V470-S660 structure (core of the P domain in Figure 4C) of the ORF2 protein of genotype 3b HEV (JE03-1760F) using the AlphaFold2 software program (Figure 4D). The P domain of the predicted structure almost matched with the previously reported HEV VLP comprising ORF2 structures, 3HAG (genotype 4) [16], 2ZTN (genotype 3) [18], and 6LAT (genotype 1) [27] (Figure 5). The C-terminal disordered region in the previous study [17] was predicted to be four α-helices and loop conformations (Figure 4D and Figure 5). In Figure 4D, the highlighted amino acids in red and cyan are the predicted trypsin digestion site, R578, and the predicted α-chymotrypsin digestion site, L601, respectively, with amino acid sequences of V579-S660 (yellow) and A574-G576 (green). The V579-L601 has two β-strands interacting with β-sheets (β-barrel; Figure 4D), which are possibly unstable because of trypsin digestion.

Our results thus suggest that the HEV virion-associated ORF2 proteins are digested with trypsin, elastase, and α-chymotrypsin, and that they lack at least A602-S660, the region with four α-helices connected by fluctuating loops, and also possibly V579-L601.

### 2.3. The ORF2 Protein of Rat HEV in Feces Is C-Terminally Truncated, Similar to That of the Fecal HEV in Humans

**Figure 6:**
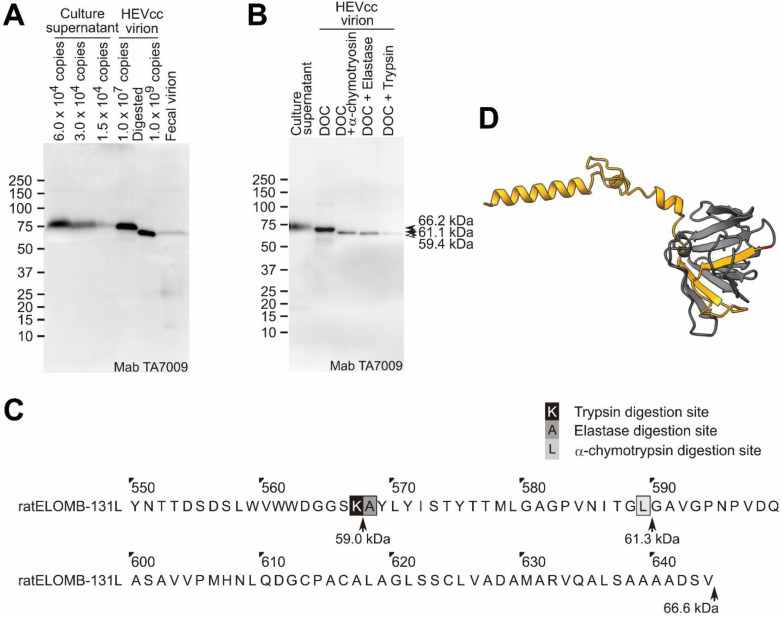
Molecular size of the ORF2 protein of rat HEV in feces. The truncated rat ORF2 protein in the rat fecal sample and the protease-digested rat HEV virion. (**A**) The culture supernatant (4.8, 2.4, and 1.2 µL of culture supernatant, containing 6.0, 3.0, and 1.5 × 10^4^ copies of HEV, respectively, per lane), the ultracentrifuged HEVcc virion (1.0 × 10^7^ copies of HEV, derived from 800 µL of culture supernatant/lane), the 0.5% DOC- and 0.5% trypsin-treated ultracentrifuged HEVcc virion (1.0 × 10^9^ copies/lane: digested), and the ultracentrifuged nonenveloped HEV virion (1 × 10^9^ copies/lane: fecal virion) in the fecal sample from rat HEV (ratELOMB-131L)-inoculated immunodeficient rats were subjected to SDS-PAGE, and were detected with the antirat HEV mouse monoclonal antibody TA7009. The estimated molecular weights of the ORF2 proteins were approximately 67 kDa for both the 0.5% DOC- and 0.5% trypsin-treated HEVcc and the fecal HEV. (**B**) The culture supernatant (1.2 × 10^4^ copies of HEV, derived from 1.2 µL of culture supernatant/lane), the ultracentrifuged HEVcc virion (1.0 × 10^7^ copies of HEV, derived from 1.0 mL of culture supernatant/lane), the 0.5% DOC-treated and 0.5% α-chymotrypsin-digested, 0.5% DOC-treated and 0.5% elastase-digested, and 0.5% DOC-treated and 0.5% trypsin-digested HEVcc virions were subjected to SDS-PAGE, and were detected with the anti-HEV mouse monoclonal antibody, TA7009. Arrowhead, arrow, and hollow arrow indicate full-length ORF2 (66.2 kDa), α-chymotrypsin-digested ORF2 (61.1 kDa), and trypsin- or elastase-digested ORF2 proteins (59.4 kDa), respectively. Molecular weights were estimated with the size marker. (**C**) The amino acid sequence of the Y550-V644 amino acids of the rat HEV ORF2 protein. Arrows indicate the calculated molecular weights: K567, 60.6 kDa; L589, 62.7 kDa; and V644, 69.3 kDa. (**D**) The structure of the P domain and C-terminal region (I451-V644) of the rat ORF2 protein structure was predicted with the AlphaFold2 software program. The K567 residue and A568-V644 residues are highlighted in red and yellow, respectively.

To investigate whether or not the ORF2 proteins of rat HEV in feces are also C-terminally truncated, we prepared a fecal rat HEV suspension and subjected it to Western blotting. The molecular size of the fecal rat HEV-associated ORF2 protein was 67.3 kDa, which is similar to that of the 0.5% DOC-treated and 0.5% trypsin-digested ORF2 protein of rat HEVcc (Figure 6A). The typical proteases secreted in the intestine, trypsin, elastase, and α-chymotrypsin truncated the ORF2 protein of rat HEVcc (Figure 6B), and were estimated to digest it at residues K567, A568, and L589, respectively (Figure 6C). The K567 residue of the ORF2 protein is conserved among various rat HEV strains (Supplementary Table S2).

To visualize the trypsin digestion site, we predicted the structure of the rat HEV (ratELOMB-131L) ORF2 protein using the AlphaFold2 software program. The two β-strands followed by the trypsin digestion site, K567, highlighted in red and interacting with other β-sheets, were considered unstable because of the trypsin digestion (Figure 6D). Thus, the three α-helices at the C-terminal end, and possibly the two β-strands in the P domain of the rat HEV ORF2 protein, were considered to be lost in the feces, similar to the findings of the fecal HEV virion in humans.

These data collectively indicate that the ORF2 proteins associated with rat HEV in feces, and those of rat HEVcc treated with detergent and protease, are C-terminally truncated (ORF2tr).

### 2.4. The Predicted Structures of Nondigested and Digested HEV Virions

**Figure 7:**
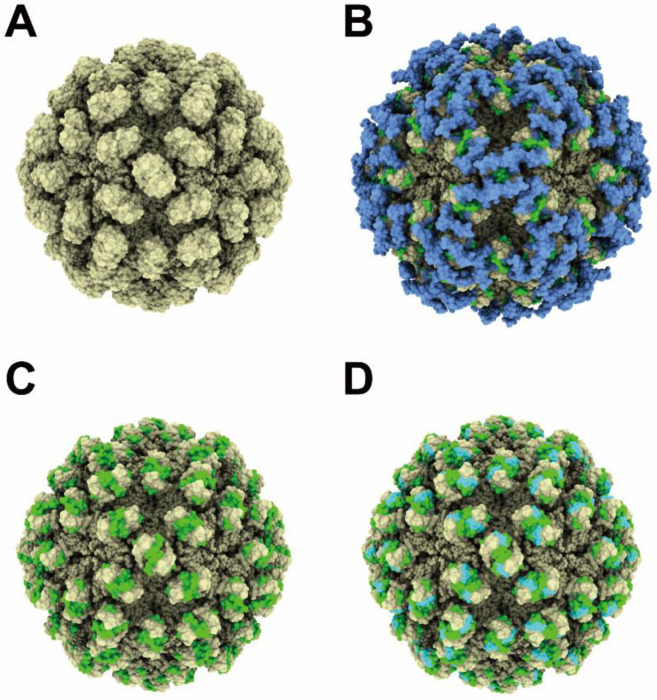
Protease-digested HEV virion structure. A predicted nonenveloped and nondigested HEV and nonenveloped and trypsin-digested virion structure. (**A**) Model-fitting image of the previously reported X-ray model of *T* = 3 VLP (PDB ID: 3IYO). The V470-A606 is replaced by the V470-A606 of the AlphaFold2-predicted ORF2 structure. (**B**) Superimposed image of 3IYO and the AlphaFold2-predicted ORF2 structure. The V470-A606 is replaced by the V470-S660 of the AlphaFold2-predicted ORF2 structure. The H6225-antibody-recognizing region, G551-R578, and the C-terminal region cleaved by trypsin V579-S660 are highlighted in green and blue, respectively. (**C**) The H6225-antibody-recognizing region, G551-R578, is highlighted in green. (**D**) The G551-R578 region and the V579-L601 region, which may be retained on the surface of fecal HEV or HEVcc, after treatment with detergent and trypsin, because of the interaction between the two β-strands (579–582; and 595–600) and other β-sheets (492–495 and 568–572; and 470–476 and 546–549, respectively), are highlighted in green and cyan, respectively.

Previously, Xing et al. [17] reported the X-ray model of a *T* = 3 icosahedral VLP, containing the HEV RNA genome fragment, with a cryo-electron microscope (cryo-EM, PDB ID: 3IYO; aa 118–606). Because the AlphaFold2-predicted ORF2 protein P-domain structure is almost the same as the previously reported ORF2 protein P domains (Figure 5), we replaced the P domain, V470-A606, of the 3IYO, with the V470-A606 of the AlphaFold2-predicted ORF2 protein structure, as 3IYO consists of only Cα atoms (Figure 7A). We then superimposed this VLP and the AlphaFold2-predicted ORF2 structure (Figure 4D) as a predicted de-enveloped and nondigested HEV virion. The superimposed AlphaFold2-predicted ORF2 protein structures (V470-A606) almost matched with those of 3IYO (data not shown). The A602-A606 in Figure 7A was replaced with the A602-S660 of the AlphaFold2-predicted ORF2 (Figure 7B). In addition, we displayed the de-enveloped trypsin-digested HEV virion structure, which lost V579-S660 (highlighted in blue in Figure 7B). The H6225 Mab recognizes the region (highlighted in green) on the surface of the predicted trypsin-digested virion (Figure 7C). This predicted model supports the finding that H6225 Mab can capture fecal HEV [28]. Therefore, it is likely that the predicted HEV virion (Figure 7B) is digestible by proteases into smaller particles (Figure 7C), which H6225 Mab is able to capture, indicating that the smaller particles (Figure 7C) contain the H6225 Mab recognition site of the ORF2 protein (G551-A608).

## 3. Discussion

The present study reveals that the molecular size of both the human and rat ORF2 proteins associated with the fecal HEV virions (ORF2tr) were smaller than the ORF2 proteins (aa 14/16–660 [20,26] in human HEV, and probably aa 21 [2 methionine]–644 in rat HEV) associated with the enveloped HEVcc virions. Sodium deoxycholate and typical proteases, including trypsin, elastase, and α-chymotrypsin, all secreted in the intestine, were found to de-envelope and digest enveloped HEV virions into smaller nonenveloped particles consisting of ORF2tr proteins. The structural prediction study on the human HEV ORF2 protein suggests that at least the A602-S660 fragment, which has four α-helixes and does not participate in the structure of the P domain, is dissociated from the HEV virions in feces.

In this study, to generate an HEV-like particle with a FLAG-tagged ORF2 protein, we inserted the nucleotide sequence corresponding to the FLAG tag (DYKDDDDK) and the 7092–7151 (60 bases) repeat into the parental genotype 3b HEV JE03-1760F/P10 genome (Figure 3A). One stem-loop structure of the *cis*-reactive element (CRE) overlaps with the 3′-end of the ORF2-coding region and the 3′-terminal sequence [29]. Because the insertion of the FLAG-tag sequence disrupted the secondary structure harboring the CRE, the HEV-like particle, with the genome having the FLAG-tagged ORF2 sequence but not the 7092–7151 repeat, did not grow in cell culture (Figure 3B). These results corroborate previous findings by confirming that the secondary structure harboring the CRE in the 3′-terminus is important for replicating the HEV genome [29,30,31]. In addition, we inserted the EGFP sequence into the JE03-1760F/P10 HEV RNA genome instead of the FLAG tag sequence, together with the 7092–7151 repeat, and transfected the recombinant JE03-1760F/P10 genome into cultured cells, and then monitored the growth of the EGFP-tagged HEV-like particle. Contrary to our expectation, the EGFP-tagged HEV-like particle was not detectable, and even the complete revertant without the EGFP genome sequence was generated in the cell culture (Supplementary Figure S1). These data indicate that the addition of an extra sequence is limited at the 3′-terminal end of the ORF2 coding sequence.

Trypsin, elastase, and α-chymotrypsin secreted in the intestine digest the ORF2 protein associated with the HEVcc virion at different sites because of the differences in their recognition sites (Figure 4A,B), especially the trypsin digestion site is R578, which is inside a core of the P domain. In a previous study [16], A574-G576 was reported to be a fluctuating region, according to a cryo-EM analysis (Figure 5), which suggests that trypsin is able to recognize and digest the downstream R578. Among the three typical proteases secreted in the intestine, trypsin digests ORF2 proteins at the C-terminal region into the smallest size, and the resulting protein (ORF2tr) was estimated to have the same size as the ORF2tr protein of the fecal HEV virion (Figure 1). However, in contrast to the ORF2tr, the glycosylated and dimerized ORF2 protein in the culture supernatant was reported to be digested into a smaller size than the ORF2tr protein [35]. Therefore, it is very likely that the DOC-treated trypsin-digested HEVcc virion possesses the same characteristics as fecal HEV.

Xing et al. [17] reported the X-ray model of *T* = 3 icosahedral VLP containing an HEV RNA fragment with a cryo-EM (PDB ID: 3IYO). This model contained an ordered structure with D118-A606. We superimposed 3IYO and the AlphaFold2-predicted ORF2 structure to visualize the nonenveloped and nondigested HEV virion (Figure 7B). Because AlphaFold2 predicted that L607-S660 would fluctuate in 3IYO, the four α-helices at the C-terminal region were expected not to constitute the ordered domain structure. In addition, we previously reported that H6225 Mab was able to capture the DOC-treated nondigested HEV virion in circulating blood [25] and culture supernatant [23]. Furthermore, the α-helices at the C-terminal end were predicted to move vertically with the WebNMA server [36] (available at: http://apps.cbu.uib.no/webnma3/, accessed on 6 December 2021, Figure 8), while other models predicted with WebNMA indicated that the α-helices move horizontally (data not shown); thus, these α-helices were considered to move freely on the surface of the HEV virion. Since the α-helices are fluctuating, a structural analysis (such as X-ray crystallography and NMR) is unable to determine the unique structure of the HEV virion consisting of full-length ORF2 protein. Only structure prediction is able to predict the HEV structure consisting of the full-length ORF2 proteins. Therefore, the combination structure involving the HEV VLP, reported by Xing et al. [17], and our structure, predicted with AlphaFold2, displayed in Figure 7B–D, has important implications in structural studies and interaction analyses.

In the present study, the ORF2 protein constituting the fecal HEV virion was estimated to be cleaved at R578/V579 and L601/A602 by trypsin and α-chymotrypsin, respectively. The extreme C-terminal end fragment of A602-S660 seems to have been removed from the surface of the fecal HEV virion, with the following reasons cited: First, this region was not ordered by cryo-EM [17], suggesting that this region does not interact with the surface of the fecal HEV virion after cleavage; second, another anti-ORF2 Mab (H6210) [28], which is mapped at the V609-S660 C-terminal region of the ORF2 protein (Supplementary Figure S2), was not able to capture the fecal HEV virion [28]. In contrast, whether or not the V579-L601 fragment is retained on the virion surface in feces remains unclear. In either case, the G551-R578 region is exposed at the surface of the fecal HEV virion (Figure 7C,D), consistent with the evidence that the H6225 Mab can almost wholly capture the HEV in feces [25,28], and that this Mab recognizes not only the G551-A608 region of the ORF2 protein (Figure 2), but also the C-terminally trypsin-digested ORF2 protein associated with HEV virion, being devoid of V579-S660 (Figure 4A,B).

However, there is a possibility that the V579-L601 region may be retained on the surface of fecal HEV virion (Figure 7D) because of the interaction between the two β-strands and other β-sheets (β-barrel, Figure 4C). Previous studies support this possibility. Arias et al. [37] found in their review that, similar to HEV, human astrovirus (HAstVs) virion is digested by extracellular trypsin during maturation. The 70-kDa immature capsid protein (VP70) that constitutes the immature virion is digested into VP34, VP27, and VP25. The cleaved VP27 and VP25 bind to the mature HAstVs, consisting of VP34, and function as spike proteins. Schofield et al. [38] reported that the Mabs, whose epitopes are mapped at the R578-L607 region of genotype 1 HEV, SAR-55, neutralize the infection of fecal SAR-55 HEV to rhesus monkeys, suggesting the presence of the cleaved R578-L607 fragment. In both cases, the core of the P domain, which is expected to be a spike because of its attachment activity to HEV-susceptible cells [18], is exposed at the surface of the fecal HEV virion, since the A602-S660 region is removed from the surface of the fecal HEV virion.

During the HEV lifecycle, the C-terminus of the HEV ORF2 protein and its digestion play an important role in the virion assembly and infection of nonenveloped HEV. Nonenveloped HEV harboring an intact C-terminus, M16-S660 (ORF2^c^) [26], and/or L14-S660 (ORF2_i_) [20], is produced in infected cells. Shiota et al. [39] reported that the 52 C-terminal amino acids (V609-S660) of the ORF2 protein of genotype 3 HEV are essential for HEV encapsulation and viral particle stabilization. In our study, the C-terminal region (at least A602-S660) of the ORF2 protein was truncated in nonenveloped HEV by the digestion of proteases. Kalia et al. [40] reported that the C-terminal-truncated VLP (aa 112–607) binds to heparan sulfate proteoglycans (HSPGs) on the cell surface. Whether or not the nonenveloped HEV virion composed of ORF2 proteins with an intact C-terminus binds to HSPGs is unclear at present. In summary, once an HEV virion with ORF2 proteins with an intact C-terminus, and that is coated with the host envelope is produced, it becomes stable. The enveloped HEV is then secreted into the intestine via the biliary tract, and de-enveloped and protease-digested into nonenveloped HEV with C-terminally truncated capsid proteins (ORF2tr). The nonenveloped HEV binds to HSPGs on the surface of the HEV-susceptible cells. However, the putative receptor for nonenveloped HEV is still unknown. On the basis of our finding, that detergent-treated and protease-digested HEVcc shares the same morphological characteristics as fecal HEV, we suggest that future studies be conducted using detergent-treated and protease-digested HEV, mimicking both human and rat fecal HEV, in order to reveal the HEV lifecycle, including the details of virus attachment and internalization.

In conclusion, the present study clarified that both the human and rat ORF2 proteins associated with enveloped HEV have an intact C-terminus, but these ORF2 proteins associated with nonenveloped HEV are C-terminally truncated (ORF2tr). The typical proteases secreted in the intestine digest the virion-associated ORF2 protein into a smaller one, similar to the ORF2tr protein associated with fecal HEV. A structure prediction analysis of the ORF2 protein supported the notion that the C-terminal region of both the human and rat HEV ORF2 proteins are removed by protease digestion. A structural study of the HEV virion shape indicated that the four α-helices (A602-S660) move freely on the surface of the HEV virion; however, at least these four α-helices are removed from the surface of nonenveloped protease-digested HEV and fecal HEV. On the basis of the findings from the present study concerning the nature of the HEV virion-associated ORF2tr proteins, we conclude that studies on nonenveloped HEV virions can be performed using DOC- and protease-treated HEVcc virions when fecal samples containing HEV virions are not available. Our study is the first of its kind regarding the maturation of a nonenveloped HEV and has important ramifications in the study of the nonenveloped HEV infection mechanism.

## 4. Materials and Methods

### 4.1. Fecal Suspensions Containing Human and Rat HEV Strains

For the human fecal sample, we used a 15% (wt/vol) suspension of feces, obtained at the acute phase, from a Japanese patient who contracted a domestic infection of genotype 3b HEV (JE03-1760F), which was successfully used for the development of a cell culture system for HEV [41]. For the rat fecal sample, we used a 15% suspension of feces obtained from a nude rat (F344/NJcl-rnu/rnu (CLEA Japan, Inc., Tokyo, Japan)) experimentally infected with a rat HEV strain (ratELOMB-131), which was successfully used to develop a cell culture system for rat HEV [42]. A 15% fecal suspension in 10 mM phosphate-buffered saline, with a pH of 7.5 (PBS), was centrifuged at 6200× *g* for 5 min at 4 °C, and the resulting clear supernatants were aliquoted and stored at −80 °C until use. They were then thawed on ice, filtrated through microfilters with a pore size of 0.2 mm (Millex-GV; Merck Millipore, Billerica, MA, USA), and subjected to assays.

### 4.2. Antibodies

The anti-ORF2 monoclonal antibodies (Mabs), H6225 and H6210 (for human HEV) and TA7009 (for rat HEV), were previously raised against the recombinant ORF2 proteins (aa 111–660 of human HEV ORF2, and aa 101–644 of rat HEV ORF2, respectively) [28,43]. The anti-FLAG M2 magnetic beads were purchased from Merck Millipore.

### 4.3. Cell Culture and Inoculation

PLC/PRF/5 cells (ATCC No. CRL-8024; American Type Culture Collection, Manassas, VA, USA) were cultured as previously described [41]. For inoculation, naïve cells were seeded at 1.0 × 10^5^ cells/well onto a 24-well plate (BioLite 24 Well Multidish; 930186, Thermo Fisher Scientific, Waltham, MA, USA). The next day, the cells were washed and inoculated with a cell-culture-adapted genotype 3b HEV strain (JE03-1760F, passage 26; 1.0 × 10^5^ copies/well) as inoculum. The inoculated cells were rinsed with phosphate-buffered saline (PBS) five times to thoroughly wash out the remaining HEV and were then grown in growth medium. As described previously, half of the culture medium was exchanged for fresh growth medium every other day [44].

### 4.4. Preparation of Detergent- and/or Protease-Treated HEV Virions

The human and rat HEV in the culture supernatant and the fecal suspensions [42,45] were centrifuged at 150,000× *g* (Optima TLX; Beckman Coulter Inc, Miami, FL, USA). The resulting pellets of HEV virions were treated with 0.5% DOC, and digested with 0.5% trypsin, 0.5% elastase (Fuji Film Wako Pure Chemical Co., Tokyo, Japan), or 0.5% α-chymotrypsin (Fuji Film Wako Pure Chemical Co.), for 1 h at 37 °C. The digested HEV virions were then treated with the serine protease inhibitor, p-APMSF (Merck Millipore).

### 4.5. Immunoblotting

The cells were lysed with 2% Nonidet P-40 (NP-40) in PBS. The collected culture supernatants, cell lysates, and virions in ultracentrifuged pellets were heated to 70 °C for 10 min in SDS sample buffer (final concentration, 60 mM Tris-HCl, 2% SDS, 5% glycerol, 0.2% bromophenol blue [BPB], 5% 2-mercaptoethanol [2-ME], and 100 mM dithiothreitol [DTT]). They were separated by SDS-PAGE on an 11/17.5% step-gradient Anderson gel [46], and then electroblotted onto Immobilon-P membranes (Millipore Corporation, Billerica, MA, USA). The human and rat HEV ORF2 proteins were detected with the H6225 or H6210 mouse antihuman HEV ORF2 protein Mabs [28], or the TA7009 mouse antirat ORF2 protein Mab [43], respectively. The bound primary antibody was detected with horseradish peroxidase (HRP)-conjugated secondary antibody (#8125; Cell Signaling Technology, Danvers, MA, USA) and SuperSignal West Femto (Thermo Fisher Scientific) HRP substrate.

### 4.6. Construction of N- and/or C-Terminally Truncated ORF2 Protein and FLAG-Tagged HEV-Like Particles

To construct ORF2 protein-expressing recombinant plasmids, we inserted the nucleotide sequence of the JE03-1760F/P10 genome [47] corresponding to the ORF2 protein, which was amplified using specific primers (Supplementary Table S3), into the pCI mammalian expression plasmid vector (Promega, Madison, WI, USA) using the NheI-XbaI sites. To construct the N- and C-terminally truncated ORF2 112-350-FLAG construct, the NheI-112-350-XbaI-FLAG-NotI was amplified and inserted to the NheI-NotI sites of pCI plasmid (pCI-112-350-FLAG). To construct other N- and/or C-terminally truncated ORF2 protein expression plasmid vectors, NheI-112-608-XbaI, NheI-112-350-XbaI, NheI-151-380-XbaI, NheI-201-450-XbaI, NheI-251-500-XbaI, NheI-296-550-XbaI, NheI-336-608-XbaI, and NheI-401-660-XbaI of ORF2 were amplified with specific primers (Supplementary Table S3), and then switched with the NheI-112-350-XbaI of pCI-112-350-FLAG using the NheI-XbaI sites, and were inserted into a pCI plasmid vector using the NheI-XbaI site. The plasmid vectors were transfected to PLC/PRF/5 cells using TransIT^®^-LT1 Reagent (TAKARA, Kyoto, Japan).

The nucleotide sequence corresponding to the FLAG tag was inserted into the C-terminal end of the ORF2 coding sequence of an infectious full-length HEV cDNA clone (pJE03-1760F/P10) [47]. Furthermore, the 7091–7151 sequence of the JE03-1760/P10 genome (60 bases, 3′ end of the ORF2 coding region) was inserted after the FLAG peptide coding sequence to maintain the RNA secondary structure in the 3′-terminal sequence, containing two stem-loop structures of *cis*-reactive elements (CREs) in the HEV genome (P10_ORF2-FLAG + 60r) [30]. In addition, the FLAG-tagged ORF2 coding sequence without the 7092–7151 (60 bases) repeat RNA sequence was constructed (P10_ORF2-FLAG). As positive and negative controls for viral growth, the parental JE03-1760F/P10 (P10-wt) and JE03-1760F/P10-GAA (P10-GAA) with a GAA mutation in the ORF1 protein (as an amino acid sequence: 1560GDD1562 to GAA of JE03-1760F/P10) were used, respectively. We fused the DNA fragments amplified with specific primers (Supplementary Table S3) to construct these plasmids using an In-Fusion HD cloning kit (Z9633N; TaKaRa Bio Inc., Shiga, Japan), according to the manufacturer’s protocol.

### 4.7. In Vitro Transcription, Capping, and Transfection

P10-wt, P10-GAA, P10_ORF2-FLAG, or P10_ORF2-FLAG+60r plasmids were linearized with BamHI-HF (R0136; New England Biolabs Inc., Ipswich, MA, USA). The linearized plasmids were subjected to the synthesis of P10-wt, P10-GAA, P10_ORF2-FLAG, and P10_ORF2-FLAG+60r RNA with an AmpriScribe™ T7-FlashTM Transcription Kit (ASF3507; epicentre/Illumina, Inc., San Diego, CA, USA), and were then purified and capped with a ScriptCap™ m7G capping system (C-SCCE0625; CELLSCRIPT, Madison, WI, USA), according to the manufacturer’s protocol. The synthesized RNAs were transfected to PLC/PRF/5 cells with a TransIT-mRNA Transfection Kit (MIR2225; Mirus Bio LLC., Madison, WI, USA), according to the manufacturer’s protocol.

### 4.8. Quantitation of HEV RNA

RNA was purified using TRIzol-LS Reagent (Thermo Fisher Scientific) from the culture supernatant, according to the manufacturer’s protocol. HEV RNA was then quantitated by quantitative reverse transcription polymerase chain reaction (RT-qPCR) with a 7900HT Fast Real-Time PCR System (Applied Biosystems, Foster City, CA, USA) using specific primers, a TaqMan probe set targeting the ORF2 and ORF3 overlapping region [28], and a QuantiTect Probe RT-PCR Kit (Qiagen, Hilden, Germany), according to the manufacturer’s protocol.

### 4.9. The Pull-Down Assay

The FLAG-tagged HEV-like particles were treated with 0.5% DOC for 1 h at 37 °C to remove the envelope. The HEV-like particles were then pelleted and resuspended in Tris-EDTA (TE) buffer (pH 7.6), supplemented with 150 mM NaCl (TEN). Anti-FLAG M2 magnetic beads were added to the HEV virion suspension, and then incubated for 2 h at 4 °C. The magnetic beads were rinsed in TEN and treated with 0.5% DOC only, 0.5% trypsin only, 0.5% DOC and 0.5% trypsin, or 100 mM glycine-Cl (pH 3.5). The genomic RNA of the eluted HEV-like particles was quantified by RT-qPCR.

### 4.10. Immunofluorescence

The PLC/PRF/5 cells, transfected with the ORF2 (full-length), or the FLAG-tagged ORF2 deletion mutant expression plasmids, were rinsed with PBS and fixed with 4% paraformaldehyde/PBS (paraformaldehyde: TAAB Laboratory Equipment Ltd., Reading, UK). The cells were then permeabilized with 0.2% NP-40/PBS (NP-40, N-6507; Sigma-Aldrich, St. Louis, MO, USA)) and rinsed with PBS. After that, the cells were blocked with 2% BSA/PBS (BSA, A4503; SIGMA) and probed with H6225 or H6210 Mabs, followed by Alexa-488-conjugated secondary antibody.

### 4.11. HEV Virion Structure Prediction

The Cα atoms of the previously reported ORF2 structure (3IYO) are displayed as spheres, with a radius of 3.5 Å, using the UCSF ChimeraX software program, ver. 1.1 (https://www.rbvi.ucsf.edu/chimerax/; accessed on 6 December 2021). The S (shell, 118–313), M (middle, 314–453), and P (protruding, 455–end) domains are highlighted in blue, green, and orange, respectively.

The human and rat HEV ORF2 protein structures were predicted on the basis of their full-length amino acid sequences, using the AlphaFold2 software program [48]. The predicted human HEV ORF2 structure was superimposed with the previously reported ORF2 structure, 3HAG (genotype 4) [16], 2ZTN (genotype 3) [18], and 6LAT (genotype 1) [27].

In addition, the predicted human HEV ORF2 structure was superimposed on the corresponding region of the chains A, B, and D in the *T* = 3 icosahedral VLPs (PDB ID: 3IYO) [17], using the UCSF Chimera software program, ver. 1.15 (https://www.cgl.ucsf.edu/chimera/; accessed on 6 December 2021). The V470-A606 of 3IYO was replaced by the V470-S660 of the superimposed human HEV ORF2. The Cα atoms of the D118-S469 of 3IYO are displayed as spheres, with a radius of 3.5 Å, using the UCSF ChimeraX software program, ver. 1.1. The amino acid residues, V470-S660, of the superimposed human HEV ORF2 are displayed as surface representations, using the UCSF ChimeraX software program, ver. 1.1. G551-R578 and V579-S660 are highlighted in green and blue, respectively.

## Figures and Tables

**Figure 8:**
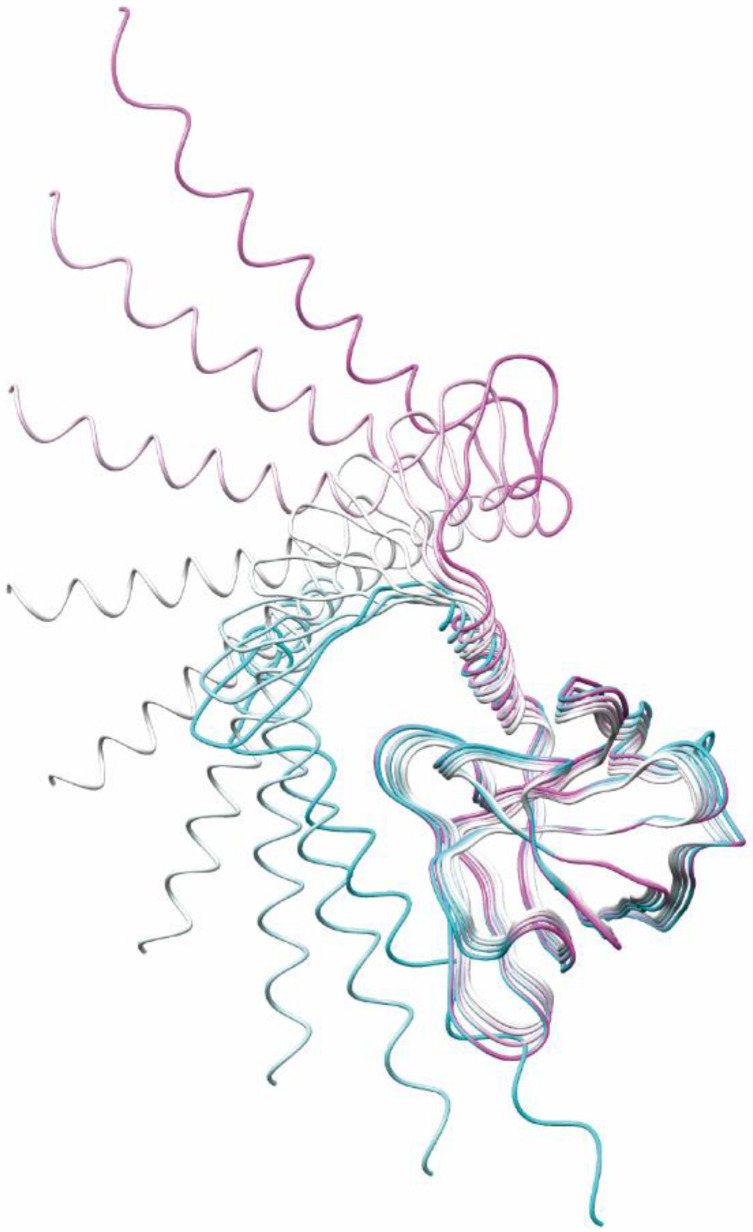
Visualization of the ORF2 protein backbone dynamics. The movement of the V470-S660 of the AlphaFold2-predicted ORF2 protein structure is colored magenta to cyan, determined via a normal mode analysis using the WebNMA server. The V470-L600 is superimposed using the UCSF Chimera software program, ver. 1.15. The α-helix at the C-terminal end is predicted to move perpendicular to the surface of the virion.

## Data Availability

Data and methods used in the research are presented in detail in this article. All data relevant to the study are included in the article.

## Supplementary Materials

The following are available online at https://www.mdpi.com/article/10.3390/pathogens11010024/s1, Figure S1: The growth curve of EGFP-tagged HEV, Figure S2: The epitope mapping of H6210, the anti-HEV ORF2 mouse monoclonal antibody, Table S1: The amino acid sequences around R578 of ORF2 proteins among the various HEV strains of genotypes 1-8, Table S2: The amino acid sequences around K567 of rat ORF2 proteins among the various rat HEV strains, Table S3: Primers used in this study.

## Abbreviations

HEV: hepatitis E virus; Mab: monoclonal antibody; DOC: sodium deoxycholate; VLP: virus-like particle; ORF2tr: truncated ORF2; aa: amino acids; CRE: *cis*-reactive element.
