# Supplementary material for: The Capsid (ORF2) Protein of Hepatitis E Virus in Feces Is C-Terminally Truncated
Source: Pathogens. 2021 Dec 26;11(1):24. doi: 10.3390/pathogens11010024 (PMC8779013; doi:10.3390/pathogens11010024)
Supplement: Supplementary file 1 [file pathogens-11-00024-s001.zip › 211221 Supplementary Figures.pdf]

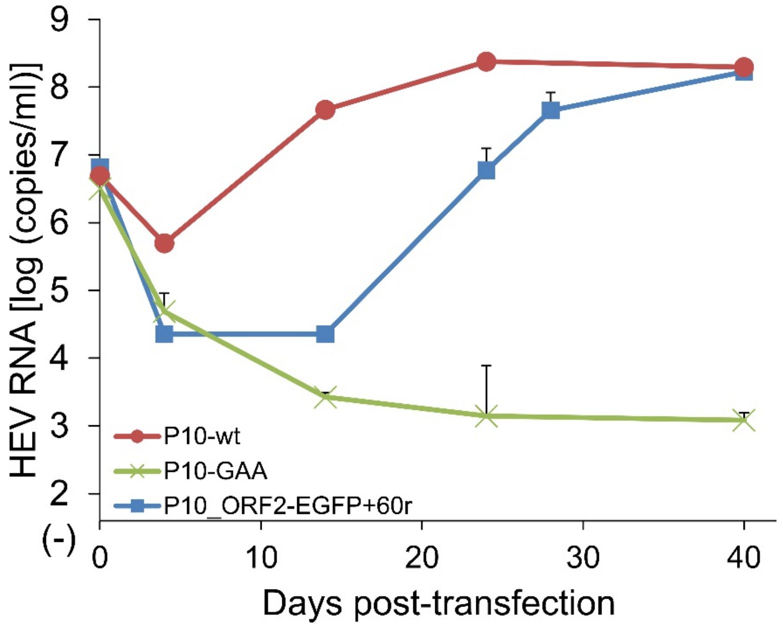

Supplementary Figure S1. The growth curve of EGFP-tagged HEV. The FLAG tag of P10_ORF2-FLAG+60r was replaced with the EGFP tag (P10_ORF2-EGFP+60r). P10-wt, P10-GAA, and P10_ORF2-EGFP+60r genome RNA were transfected to PLC/PRF/5 cells. The changes in the HEV RNA titers were monitored with RT-qPCR and plotted (n=3). Error bars represent the mean ± SD.

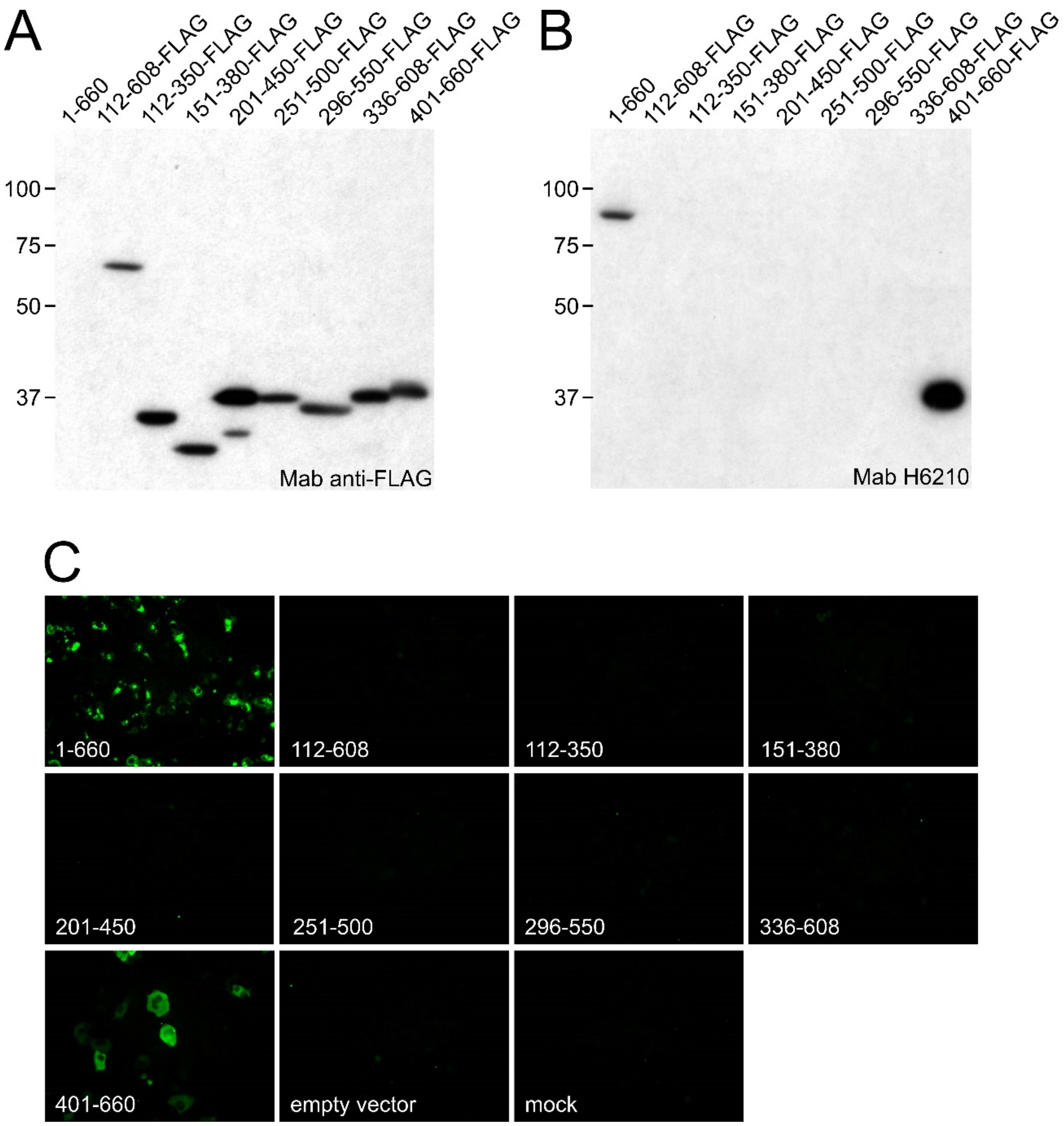

Supplementary Figure S2. The epitope mapping of H6210, the anti-HEV ORF2 mouse monoclonal antibody. The PLC/PRF/5 cells transfected into the ORF2 full-length and FLAG-tagged deletion constructs were lysed and subjected to SDS-PAGE, blotted, and then probed with anti-FLAG (A) and H6210 Mab (B). (C) Immunofluorescence analyses were performed on the transfected cells with H6210 Mab. The recognition site of H6210 Mab was mapped to the V609-S660 region of the ORF2 protein.
